# The disruption of NEAT1-miR-125b-5p-SLC1A5 cascade defines the oncogenicity and differential immune profile in head and neck squamous cell carcinoma

**DOI:** 10.1038/s41420-024-02158-1

**Published:** 2024-09-03

**Authors:** Ying-Chieh Liu, So-Yu Liu, Yu-Cheng Lin, Chung-Ji Liu, Kuo-Wei Chang, Shu-Chun Lin

**Affiliations:** 1 Institute of Oral Biology, College of Dentistry, National Yang Ming Chiao Tung University, Taipei, Taiwan; 2 Department of Dentistry, College of Dentistry, National Yang Ming Chiao Tung University, Taipei, Taiwan; 3 Department of Stomatology, Taipei Mackay Memorial Hospital, Taipei, Taiwan; 4 Department of Stomatology, Taipei Veterans General Hospital, Taipei, Taiwan

**Keywords:** Head and neck cancer, Cancer microenvironment

## Abstract

Metabolic reprogramming sustains malignant head and neck squamous cell carcinoma (HNSCC) to overcome stressful microenvironments, and increased glutamine uptake is a common metabolic hallmark in cancers. Since metabolic reprogramming has been recognized as a new therapeutic target for tumor cells, understanding the regulatory axis of glutamine uptake in HNSCC and its potential downstream effects in its pathogenesis of HNSCC would be incredibly beneficial. Bioinformatic analysis of the Cancer Genome Atlas (TCGA)-HNSCC dataset and RNAseq analysis performed on HNSCC indicated that SLC1A5 was the most dysregulated transporter among the seven homologous glutamate or neutral amino acid transporters in the SLC1A family. To further clarify the role of SLC1A5 in HNSCC, we knocked down SLC1A5 expression. This knockdown decelerated cell growth, induced G0/G1 arrest, diminished tumorigenicity, and increased cleavage caspase3, LC3B, and intracellular Fe^2+^. Inhibitors against apoptosis, autophagy, or ferroptosis rescued the cell viability repressed by SLC1A5 knockdown. SLC1A5 knockdown also suppressed glutamine uptake, enhanced oxidative stress, and increased sensitivity to cisplatin. CRISPR/dCas9-mediated SLC1A5 induction conferred cisplatin resistance and reduced apoptosis, autophagy, and ferroptosis. Reporter assays and western blot data demonstrated that miR-125b-5p targets and attenuates SLC1A5, while the si-NEAT1 increases miR-125b-5p expression. Analysis of the TCGA-HNSCC databases showed concordant upregulation of NEAT1 and downregulation of miR-125b-5p, along with SLC1A5 upregulation in tumors. Analysis of transcriptomic data revealed that tumors harboring higher SLC1A5 expression had significantly lower immune scores in CD8^+^, monocytes, and dendritic cells, and higher scores in M0 and M1 macrophages. Disruptions in immune modulation, metabolism, and oxidative stress components were associated with SLC1A5 aberrations in HNSCC. This study concludes that the NEAT1/miR-125b-5p/SLC1A5 cascade modulates diverse activities in oncogenicity, treatment efficacy, and immune cell profiles in head and neck/oral carcinoma.

## Introduction

To overcome the harsh tumor microenvironment (TME), such as oxidative stress, hypoxia, and hypo-nutrition, tumor cells reprogram metabolism to gain abilities for tumor progression, metastasis, drug resistance, and redundancy in therapeutic responses. Tumors usually modulate intrinsic factors by altering the preference of metabolic pathways or increasing specific nutrient uptake. Glutamine belongs to non-essential amino acids (NEAA) and is the most abundant residue in plasma. Glutamine participates in NEAA function, nucleotide, lipid biosynthesis pathways, and tricarboxylic acid (TCA) cycle to produce energy and to form glutathione as an antioxidant. Since these metabolic pathways are essential for sustaining cell survival, many cancers maintain their survival by regulating glutamine uptake or biosynthesis. Glutamine can be transported into cells by multiple solute carrier transporters. Solute carrier family 1 member 5 (SLC1A5), which belongs to Alanine/Serine/Cysteine transporters, transports glutamate into cells [1]. High SLC1A5 expression levels could facilitate tumor proliferation and antioxidant capacity and are associated with poor prognosis and the ability to resist dasatinib, sunitinib, sorafenib, and imatinib treatment in several malignancies [2–5]. SLC1A5 expression progressively increased during the pathogenesis of oral squamous cell carcinoma (OSCC), and it regulates glutamine metabolism or antioxidant function to mediate tumor progression [6]. Knockdown of SLC1A5 expression inhibits glutamine-addicted OSCC cell proliferation, and the SLC1A5 protein expression is an unfavorable prognostic factor in a cohort of 89 OSCC [7]. SLC1A5 inhibits apoptosis and autophagy in HNSCC. Besides, combining the treatment of glutamine uptake inhibitors with cetuximab could be a promising strategy to improve the survival of HNSCC patients [8]. However, the anti-oxidative activity of SLC1A5 in preventing ferroptotic HNSCC cell death and whether SLC1A5 expression is a prognostic factor in HNSCC remain elusive.

The expression level of SLC1A5 is correlated with the expression of immune checkpoint-related genes such as Cytotoxic T-lymphocyte associated protein 4 (CTLA4), programmed death-1 (PD-1), programmed cell death 1 ligand 2 (PD-L2), and T-cell immunoreceptor with immunoglobulin and immunoreceptor tyrosine-based inhibition motif domain (TIGIT) [9]. The high SLC1A5 expression in tumor cells may modulate tumor-infiltrating immune cells, as the knockdown of SLC1A5 expression recruits M2 macrophage infiltration to increase immunotherapy efficiency in pancreatic adenocarcinoma [5]. In the glutamine-signature-high group of clear renal cell carcinoma, hypoxia-inducible factor 1 α (HIF-1α) expression and interleukin-23 (IL-23) secretion in macrophages are observed, followed by immune suppression. Tumor growth in vivo can be inhibited by neutralizing IL-23A with antibodies or by knocking down SLC1A5 gene expression [10]. A previous study showed that the higher the expression of SLC1A5 in HNSCC tumor cells, the lower the infiltration of CD8^+^ T cells, even though SLC1A5 expression is irrelevant to patient survival [11]. Therefore, SLC1A5 is an intrinsic factor regulating tumor growth and an extrinsic factor modulating the immune profile in TME. However, the influences of SLC1A5 on the immune TME in HNSCC require extensive stratification.

Long non-coding RNAs (lncRNAs) Nuclear Enriched Abundant Transcript 1 (NEAT1) are essential for the paraspeckle formation, which is critical for gene regulation via interactions with DNA, RNA, or protein to maintain normal cellular function. NEAT1 is frequently upregulated in tumor cells. It may function as a miRNA sponge to inhibit suppressor miRNAs, which subsequently induces targeted oncogenic gene expression, such as the miR-204-5p/SEMA4B axis or the miR-377/FGFR1 axis [12–16]. It is worth noting that miR-125b-5p is a vital suppressor miRNA that can target peroxiredoxins like 2 A (PRXL2A), signal transducer and activator of transcription 3 (STAT3), matrix metalloproteinase-2 (MMP2), and others to induce tumor cell death or increase drug sensitivity [17–19]. A previous study found that NEAT1 could inhibit miR-125b-5p expression to modulate glucose-associated disease pathogenesis [20].

This study identifies NEAT1 upregulation and miR-125b-5p downregulation in TCGA-HNSCC data and in our cohort. The miR-125b-5p expression increases following si-NEAT treatment in HNSCC cells. The reporter assay and bioassays reveal that SLC1A5 is a target gene of miR-125b-5p. The NEAT1/miR-125b-5p interplay modulates SLC1A5 expression in HNSCC. The knockdown of SLC1A5 in OSCC cell lines promotes cell death, induces oxidative stress, and enhances sensitivity to cisplatin treatment. Tumors with SLC1A5 dysregulation exhibit a unique immune infiltration profile in the TME. IL6/Janus kinase/signal transducer and activator of transcription 3 (IL6/Jak/Stat3) and IL2/signal transducer and activator of transcription 5 (IL2/Stat5) axes correlate with the SLC1A5 expression. SLC1A5 modulates tumor phenotypes and immune profile, and it exhibits tremendous potential to become a therapeutic target in future HNSCC/OSCC treatment.

## Results

### SLC1A5 was upregulated in tumors and was associated with worse tumor differentiation

**Fig. 1:**
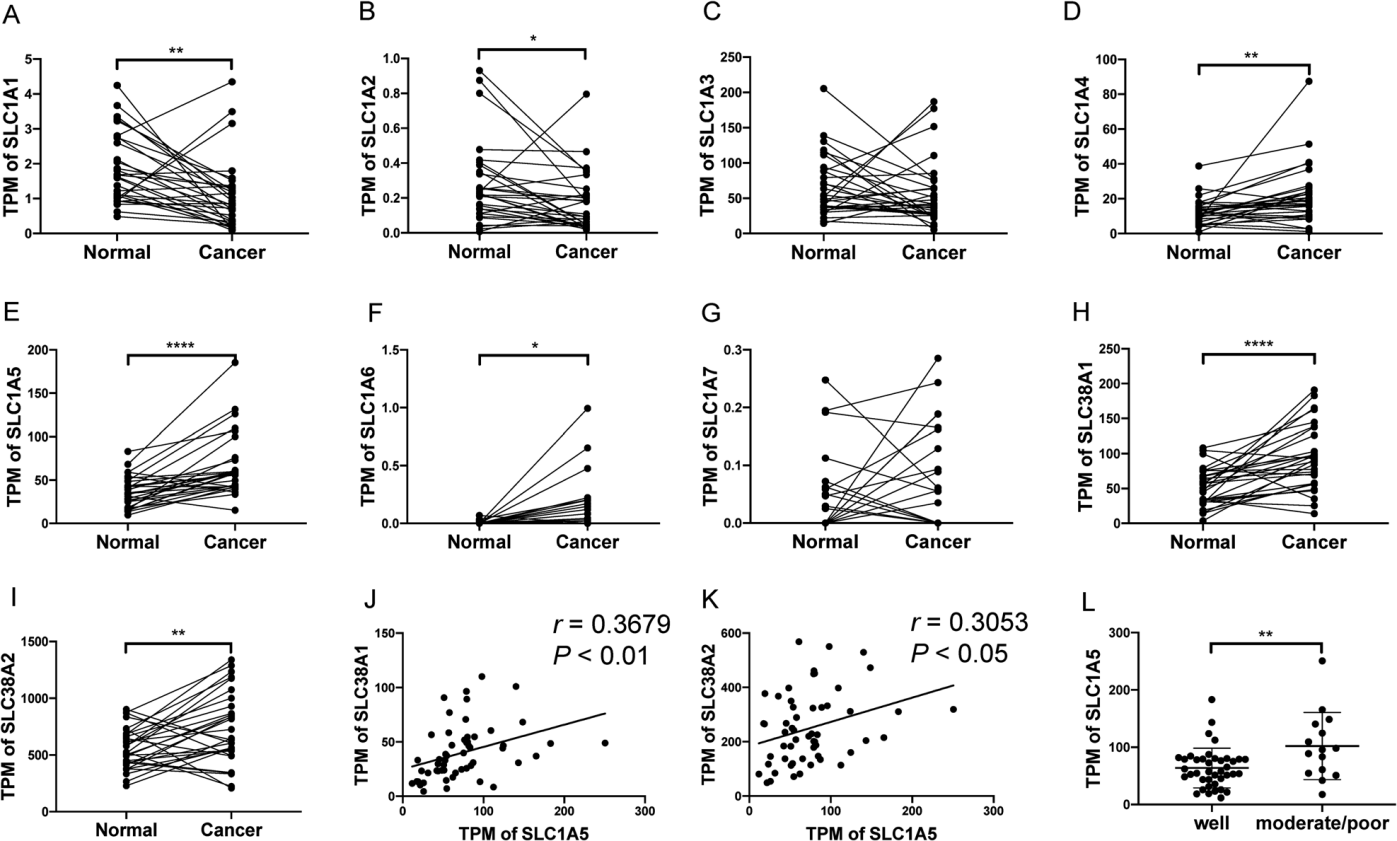
Analysis of OSCC RNA sequencing data. **A**–**I** The TPM of the SLC1A family, SLC38A1, and SLC38A2 in 30 paired adjacent normal tissues and OSCC tumor tissue. **J**, **K** The correlation between SLC1A5 and SLC38A1 or SLC38A2, respectively. **L** The correlation between SLC1A5 and tumor differentiation (well-differentiated, *n* = 40; moderately/poorly-differentiated, *n* = 15). Paired *t-*test, Mann-Whitney test, or correlation analysis. *r*, correlation coefficient. *, **, and ****, *P* < 0.05, *P* < 0.01, and *P* < 0.0001, respectively.

To understand which members of the SLC1A receptor family might have played an essential role in HNSCC pathogenesis due to aberrant expression, we analyzed the RNA sequencing data of the SLC1A family in adjacent normal control and OSCC tumors. SLC1A5 was shown to be the most conspicuously upregulated among the SLC1A receptor members. SLC1A1 and SLC1A2 were downregulated in tumor samples (Fig. 1A–G). The upregulation of SLC1A5 and SLC1A4 and the downregulation of SLC1A1 were also conspicuous in TCGA-HNSCC tumors (Fig. S1A–G). Due to the vital role of SLC1A5 in glutamine transport, we examined other SLC transporters that were also involved in glutamine uptake. Among the SLC family, SLC38A1 and SLC38A2 are also known for glutamine transportation [21]. Analysis of paired samples showed their drastic upregulation in tumors (Fig. 1H, I) and a positive correlation with SLC1A5 expression (Fig. 1J, K). Analysis of the TCGA-HNSCC dataset also indicated the copious upregulation of SLC38A1 and SLC38A2 (Fig. S1H, I). However, only SLC38A1 expression was positively correlated with SLC1A5 expression (Fig. S1J and K, Table S1). Although the dysregulation of other potential glutamine transporters, such as SLC7A5, was also present in HNSCC (Table S2), none of their expressions positively correlated with SLC1A5 expression. Therefore, SLC1A5 and SLC38A1 could have been highly concurred in modulating glutamine transport in HNSCC/OSCC.

SLC1A5 expression was higher in moderately/poorly differentiated OSCC or TCGA-HNSCC tumors than well-differentiated counterparts (Figs. 1L and S1L, respectively), suggesting that glutamine uptake might have been more demanded in tumors exhibiting less squamous differentiation. SLC1A5 expression level was not related to clinical stage, lymph node metastasis, or overall survival rate of tumors (Fig. S2).

### Knockdown of SLC1A5 expression inhibited the growth and increased the cisplatin sensitivity in HNSCC cells

**Fig. 2:**
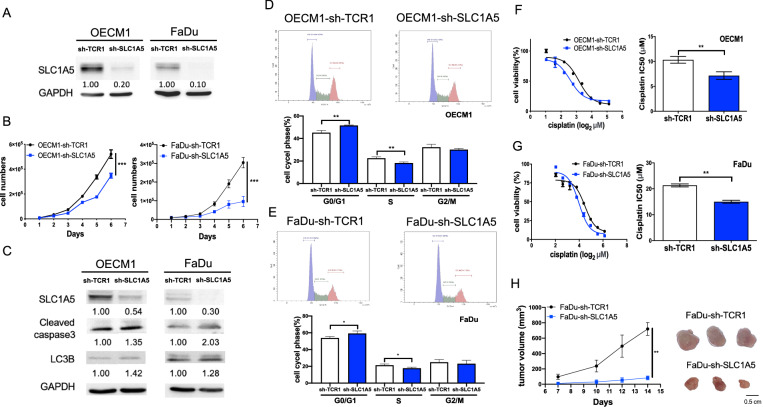
Association between SLC1A5 expression and the survival of HNSCC cell subclones. sh-TCR1, control; sh-SLC1A5, knockdown of SLC1A5. **A** Western blot analysis to reveal the knockdown of SLC1A5 expression in OECM1 and FaDu cell subclones. **B** Growth curve to designate the robust decrease of cell proliferation in SLC1A5 knockdown cell subclones. **C** Western blot analysis to reveal the increase of apoptosis (cleaved caspase-3) and autophagy (LC3B) biomarkers following the knockdown of SLC1A5. **D**, **E** Cell cycle analysis of OECM1 and FaDu cell subclones, respectively. Upper, flow cytometry diagrams, Lower, quantitation of phases. **F**, **G** Lt, OECM1 and FaDu cell subclones’ survival curves after cisplatin treatment disclosed by MTT assay. Rt, Quantitation of IC_50_. Knockdown of SLC1A5 sensitizes cells to cisplatin toxicity. **H** The in vivo tumorigenicity of FaDu cell subclones in nude mice. Lt, growth curves. Rt, the gross tumor pictures. It is composed of individual pictures with equivalent magnification. Numbers in the western blot are the normalized expression value. Mann-Whitney test or Anova analysis. *, **, and ***, *P* < 0.05, *P* < 0.01, and *P* < 0.001, respectively.

To address the functional roles of SLC1A5, SLC1A5 knockdown FaDu and OECM1 cell subclones were established by sh-SLC1A5 viral infection (Fig. 2A). As inhibition of SLC1A5 expression alone rendered a significant reduction of cell growth in cell subclones relative to control, the crucial roles of SLC1A5 in cell growth are certified (Fig. 2B). Both LC3B and cleaved caspase-3 increased in SLC1A5 knockdown cell subclones (Fig. 2C). Since the reduction in cell numbers might have resulted from the impediment of cell proliferation or the increase in cell death, we further explored the cell cycle changes and the biomarkers of cell death. In SLC1A5 knockdown cell subclones, the cell cycle was arrested in the G0/G1 phase. The cell proportion in the S phase was decreased (Fig. 2D, E). Cisplatin is a standard regimen for HNSCC chemotherapy. Half-maximal inhibitory concentration (IC_50_) analysis indicated that the SLC1A5 knockdown cell subclones were more sensitive to cisplatin treatment (Fig. 2F, G). The IC_50_ of OECM1-sh-TCR1 was 11.3 µM, while the IC_50_ of OECM1-sh-SLC1A5 was 6.9 µM (Fig. 2F). The IC_50_ of FaDu-sh-TCR1 was 22.3 µM, while the IC_50_ of FaDu-sh-SLC1A5 was 15.7 µM (Fig. 2G). In addition, the growth of FaDu xenografts with the knockdown of SLC1A5 was slower than control xenografts (Fig. 2H). The knockdown of SLC1A5 expression significantly inhibited the growth and the resistance to cisplatin in HNSCC cells.

### Association between SLC1A5 level and the ROS in cells

**Fig. 3:**
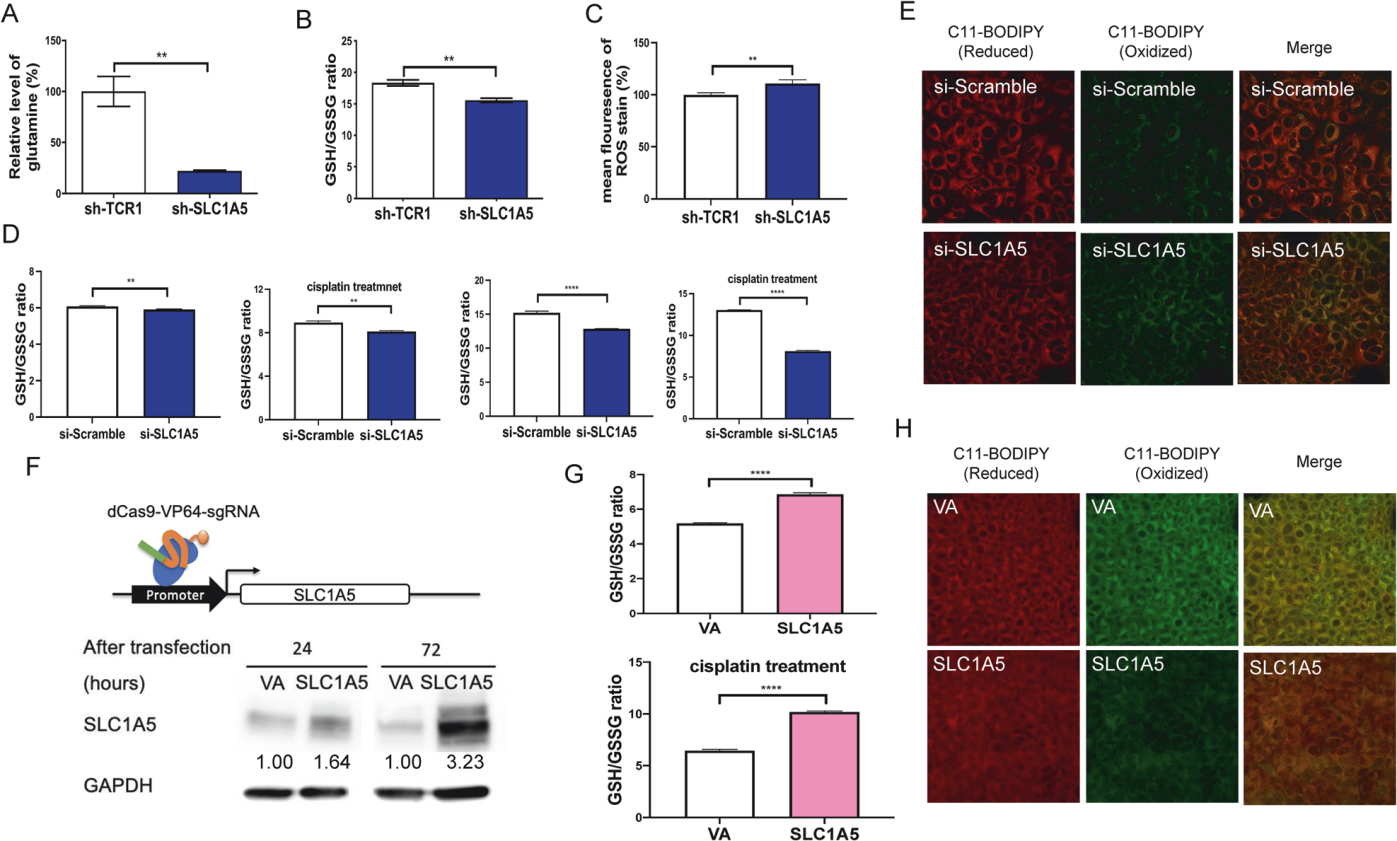
The ROS-associated states in OECM1 cells following the knockdown or induction of SLC1A5 expression. **A**–**C** The glutamine, GSH/GSSG ratio, and ROS detected by CellROX Deep Red Reagent staining in cell subclones, respectively. **D**, **E** Cells transfected with si-Scramble or si-SLC1A5. **D** The GSH/GSSG ratio. Cells treated without or with 6.9 µM cisplatin for 24 hour (let panels) or 48 hours (right panels). **E** Confocal microscopy to illustrate the C11-BODIPY-581/591 labeled polyunsaturated fatty acids in cells after 24 hours si-RNA transfection. **F** Illustration of SLC1A5 promoter region, the sgRNA targeting site, and western blot analysis. The transfection of SLC1A5-MS2-vector in OECM1-dCas9-SAM cell subclone upregulates SLC1A5 expression. Numbers designate the normalized expression value. **G** The GSH/GSSG ratio in cells transfected with VA or SLC1A5 plasmids, without (upper) or with (lower) the 6.9 µM cisplatin treatment. **H** Confocal microscopy to detect the staining of C11-BODIPY-581/591. In (**E**) and (**H**), Lt panels, reduced form [42]; Middle panels, oxidized form (green); Rt panels, merged images. VA, vector alone. Mann-Whitney test. ** and ****, *P* < 0.01 and *P* < 0.0001, respectively.

Further examination showed the decreased level of glutamine (Figs. 3A and S3A) and GSH/GSSG ratio (Figs. 3B and S3B) in SLC1A5 knockdown HNSCC cell subclones compared to the sh-TCR1 group. Besides, ROS increased in the SLC1A5 knockdown group, as shown by staining with the ROS indicator dye (Figs. 3C and S3C). To confirm the effects of SLC1A5 silencing, HNSCC cells were treated with si-SLC1A5 to knock down the expression. The si-SLC1A5 treatment decreased the GSH/GSSH ratio (Fig. 3D and S3D). In cells treated with cisplatin, the si-SLC1A5 administration also decreased the GSH/GSSH ratio. Furthermore, the staining intensity of the oxidized form of polyunsaturated fatty acids also increased by si-SLC1A5 treatment compared to controls (Fig. 3E and S3E). The OECM1 dCas9-SAM cells transfected with the sgRNA vector targeting the SLC1A5 promoter successfully induced endogenous SLC1A5 expression (Fig. 3F). Upregulating endogenous SLC1A5 expression led to an increased GSH/GSSG ratio, indicating a reduction in ROS stress (Fig. 3G). In the OECM1-dCas9-SAM cells treated with cisplatin, the group overexpressing SLC1A5 exhibited lower ROS stress. Confocal microscopic images also illustrated that SLC1A5-overexpressed OECM1-dCas9-SAM cells had lower staining intensity of the oxidized form of polyunsaturated fatty acids than the control (Fig. 3H). The findings indicate that the SLC1A5 level is associated with ROS in cells.

### SLC1A5 knockdown decreased cell survival, and inhibitors rescued the cell survival

**Fig. 4:**
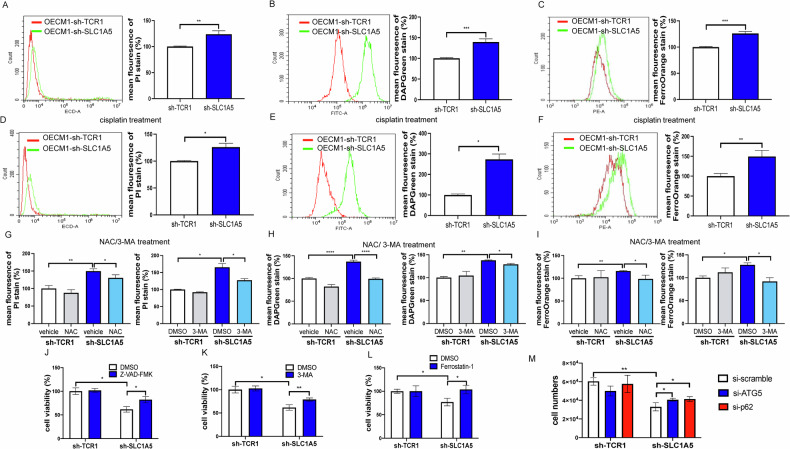
Knockdown of SLC1A5 and cell death in OECM1 cell subclones. **A**–**C** and **D**–**F** Flow cytometry analysis of cell subclones without (**A**–**C**) or with the treatment of 15.7 µM cisplatin for 48 hours (**D–F**), respectively. **A**, **D** PI stain as an indicator of apoptosis. **B**, **E** DAPGreen is the dye of autophagosome (LC3B). **C**, **F** FerroOrange is a fluorescent probe for ferroptosis (intracellular Fe^2+^). Lt, histograms, Rt, quantitation of the positive cells. **G**–**I** The quantitation of PI staining, DAPGreen staining, and FerroOrange staining in cell subclones following NAC or 3-MA. **J**–**M** Rescue assay of cell viability. Treatment with 5 µM Z-VAD-FMK (**J**), 3-MA (**K**), Ferrostatin-1 (**L**), or 60 nM si-Scramble, si-ATG5, and si-p62 (**M**) is performed in cell subclones for 48 hours, respectively. These inhibitors or siRNAs reverse the decreased cell viability associated with the knockdown of SLC1A5. Mann-Whitney test. *, ** and ***, *P* < 0.05, *P* < 0.01 and *P* < 0.001, respectively.

Glutamine is an essential nutrient for cells and a substrate for glutathione synthesis. As our assays showed a decrease in glutamine, GSH/GSSH ratio, and an increase in oxidative stress, we would like to investigate whether this also underlies cell death. An increased apoptosis, autophagy, and ferroptosis were found in the OECM1 cell subclones (Fig. 4A–C). Additionally, apoptosis and autophagy increased, but no remarkable change in ferroptosis was noted in the FaDu subclone (Fig. S4A–C). Knockdown of SLC1A5 also increased apoptosis, autophagy, and ferroptosis in cells following cisplatin treatment (Figs. 4D–F and S4D–F). The 1 mM NAC pretreatment decreased the apoptosis and autophagy in knockdown cell subclones (Figs. 4G, H Lt and S4G, H Lt), and the ferroptosis in OECM1 subclone (Fig. 4I Lt), but not in FaDu subclone (Fig. S4I Lt). The 3-MA treatment decreased the apoptosis and autophagy in knockdown cell subclones (Figs. 4G, H Rt and S4G, H Rt), and the ferroptosis in OECM1 subclone (Fig. 4I Rt), but not in FaDu subclone (Fig. S4I Rt). However, 20 mM NAC pretreatment decreased cisplatin-induced ferroptosis in the SLC1A5 knockdown FaDu subclones (Fig. S4J).

To examine if various types of programmed cell death might have underlain the decrease of cell viability, we treated cells with the apoptosis inhibitor Z-VAD-FMK (Figs. 4J and S4K), autophagy inhibitor 3-MA, (Figs. 4K and S4L) or ferroptosis inhibitor ferrostatin-1 (Figs. 4L and S4M); and silenced ATG-5 or p62 expression using siRNA delivery (Figs. 4M and S4N). The decreased cell survival secondary to the knockdown of SLC1A5 was also rescued with the treatments of these inhibitors and siRNAs.

### The upstream NEAT1/miR-125b-5p axis modulated SLC1A5 expression

**Fig. 5:**
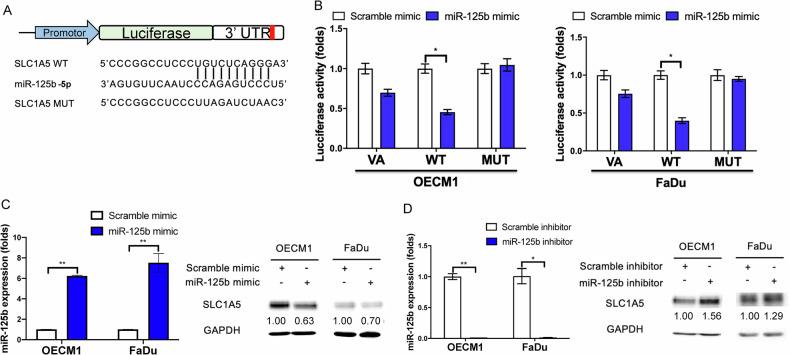
The targeting of SLC1A5 by miR-125b-5p in HNSCC cells. **A** Illustration of miR-125b-5p targeting site in SLC1A5 3’UTR. The complementarity between miR-125b-5p and SLC1A5 in wild-type reporter (WT) is abolished in the mutated reporter (MUT). **B** reporter assay by co-transfecting VA, SLC1A5 WT, and SLC1A5 MUT vector with or without miR-125b-5p mimic. **C**, **D** Lt panels, upregulation or downregulation of miR-125b-5p by transfecting mimic or inhibitor reagents. Rt panels, western blot analysis to show the decrease or increase of SLC1A5 protein expression following the treatment with mimic or inhibitor, respectively. Numbers designate the normalized expression value. Mann-Whitney test. * and **, *P* < 0.05 and *P* < 0.01, respectively.

Our previous study showed that miR-125b-5p is a suppressor miRNA downregulated in OSCC [17]. Reporter assay was used to validate the targeting relationship. The evident decrement of SLC1A5 3’UTR wild-type reporter following miR-125b-5p mimic transfection compared to Scr transfection was observed. Meanwhile, the transfection of miR-125b-5p mimic could not suppress the luciferase activity of the SLC1A5 3’UTR mutant reporter (Fig. 5A, B). Furthermore, the transfection of miR-125b-5p mimic elevated the miR-125b-5p expression levels. It decreased SLC1A5 protein expression in OECM1 and FaDu cells (Fig. 5C). In contrast, the treatment with miR-125b-5p inhibitor to downregulate miR-125b-5p expression increased SLC1A5 protein expression in the OECM1 and FaDu cells (Fig. 5D). The results substantiate the targeting of miR-125b-5p on SLC1A5 in HNSCC cells.

**Fig. 6:**
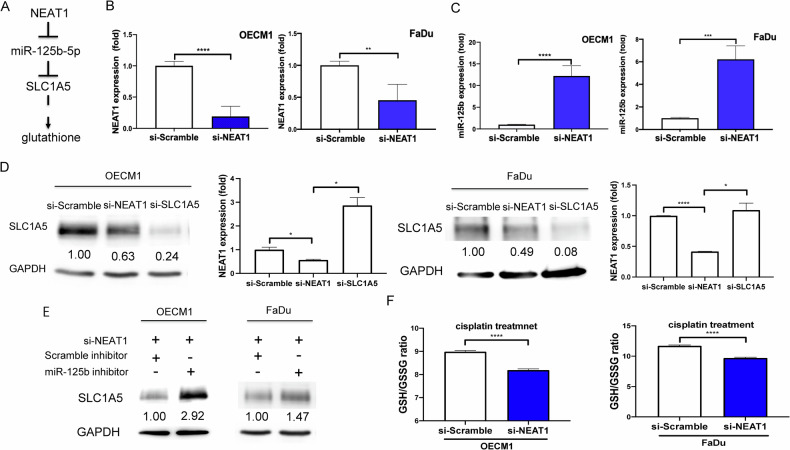
The regulatory axis of NEAT1/miR-125b-5p/SLC1A5 and the effect of NEAT1 in HNSCC cells. **A** Illustration of NEAT1/miR-125b-5p/SLC1A5 regulatory axis. **B** Knockdown of NEAT1 expression by si-NEAT1. **C** The miR-125b-5p expression fold changes after 100 nM si-NEAT1 treatment. **D** The SLC1A5 protein expression following 100 nM si-NEAT1 or 100 nM si-SLC1A5 treatment. Lt, Western blot analysis. Rt, qPCR analysis to detect NEAT1 expression. **E** The SLC1A5 protein expression following 100 nM si-NEAT1 treatment with or without the treatment with 100 nM miR-125b-5p inhibitor. Numbers designate the normalized expression value. Prolonged image exposure is done in this diagram. **F** The GSH/GSSG ratio. After the treatment with 6.9 µM or 15.7 µM cisplatin in OECM1 or FaDu cells, respectively, for 24 hours, cells are treated with si-NEAT1, and the GSH/GSSG ratios are assayed. Mann-Whitney test. *, **, *** and ****, *P* < 0.05, *P* < 0.01, *P* < 0.001 and *P* < 0.0001, respectively.

A previous study reports that NEAT1 enacts a sponge of miR-125b-5p to abrogate its activity [20]. Analysis of the database revealed the upregulation of NEAT1 in TCGA-HNSCC (Fig. S5A). To confirm the existence of the NEAT1/miR-125b-5p/SLC1A5 regulatory pathway in OSCC cells (Fig. 6A), we treated the cells with si-NEAT1 and identified the consequential increase in miR-125b-5p expression. The knockdown of NEAT1 expression using si-NEAT1 led to an elevated expression of miR-125-5p in HNSCC cells (Fig. 6B, C). The protein expression of SLC1A5 decreased with si-NEAT1 treatment (Fig. 6D). However, in the presence of si-NEAT, the SLC1A5 expression was rescued by miR-125b-5p inhibitor (Fig. 6E). Knocking down NEAT1 resulted in decrease of GSH/GSSG ratio in cells following cisplatin treatment (Fig. 6F). Despite the upregulation of NEAT1 and SLC1A5 RNA expression and the downregulation of miR-125b-5p expression in the TCGA-HNSCC database (Fig. S5A–D), no correlation in the RNA expression among these genes was found (Fig. S5E, F). However, the reporter assay, expression, inhibition and knockdown analysis substantiated the NEAT1/miR-125b/SLC1A5 interplay in vitro.

### Association between NEAT1/SLC1A5 expression and migratory phenotypes

**Fig. 7:**
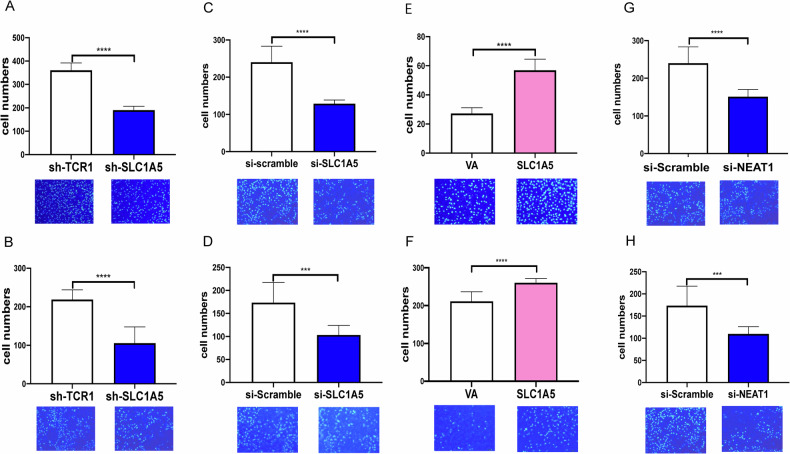
The association between SLC1A5 and the mobility phenotypes of OECM1 cells. **A**, **B** OECM1 cell subclones. **C**, **D** and **G**, **H** knockdown of SLC1A5 and NEAT1 using 60 nM and 100 nM siRNA, respectively. **E**, **F** OECM1-dCas9-SAM cells transfected with VA and SLC1A5 plasmids. **A**, **C**, **E, G** Migration assay. **B**, **D**, **F, H** invasion assay. Mann-Whitney test. *** and ****, *P* < 0.001 and *P* < 0.0001, respectively.

To examine the influence of SLC1A5 on migration and invasion, we seeded the SLC1A5 knockdown cell subclones and si-SLC1A5-treated cells in transwells. The migration and invasion abilities were decreased in the SLC1A5 knockdown cell subclones or cells treated with si-SLC1A5 relative to controls (Figs. 7A–D and S6A–D). Besides, the migration and invasion ability increased in OECM1 cells exhibiting the increased endogenous SLC1A5 expression (Fig. 7E, F). Additionally, the knockdown of NEAT1 also decreased the migration and invasion of HNSCC cells (Figs. 7G, H and S6E, F).

### Potential modulation of SLC1A5 on the tumor microenvironment and immune profile

**Fig. 8:**
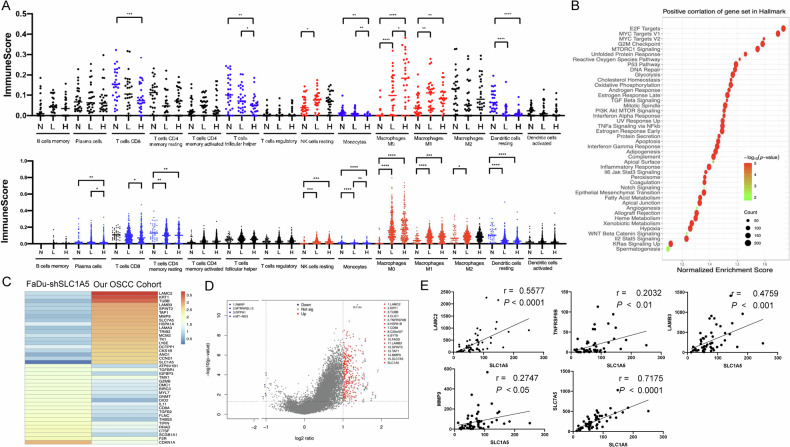
The association between SLC1A5 and immune profile in tumors. **A** Immune score of tumors analyzed by Cibersortx algorithms. Upper, OSCC tumors. Normal (N, *n* = 30), SLC1A5-Low (L, *n* = 27) and SLC1A5-High (H, *n* = 28). The medium TPM value of SLC1A5 separates tumors into L and H subsets. Lower, TCGA-HNSCC tumors. Normal (N, *n* = 43), SLC1A5-Low (L, *n* = 313) and SLC1A5-High (H, *n* = 208). The mean TPM value of SLC1A5 separates tumors into L and H subsets. Blue, decreases significantly; Red, increases significantly; Black, no statistically significant difference. **B** Comparison across SLC1A5-Low and SLC1A5-High patient groups in GSEA of cancer hallmark gene set, respectively. They highlight the conspicuous dysregulation in the immune, metabolism, oncogenicity, and cell survival according to SLC1A5 expression. **C** The fold change in the hallmark gene expression set in FaDu-sh-SLC1A5 cell subclone and our OSCC cohort. **D** Volcano plot. It deciphers genes drastically affected by SLC1A5 in OSCC tumors. The top 15 most eminently upregulated and four downregulated genes are shown in the diagram. **E** The correlation between SLC1A5 and our dataset’s top 18 upregulated genes. Herein, only five genes exhibiting significant correlation are shown. *r*, correlation coefficient. Unpaired *t*-test and correlation analysis. *, **, ***, and **** represent *P* < 0.05, *P* < 0.01, *P* < 0.001 and *P* < 0.0001, respectively.

To gain insight into the influences of SLC1A5 on the tumor microenvironment and immune profile, we analyzed RNA sequencing data of 30 normal tissues, 55 OSCC tissues, and TCGA-HNSCC datasets using the Cibersortx algorithm. In both cohorts, the scores of CD8^+^ T cells, monocytes, and dendritic cells decreased as the expression of SLC1A5 increased. On the other hand, the scores of resting NK cells, M0 macrophage, and M1 macrophage increased following the increase in SLC1A5 expression (Fig. 8A). The decrease of T follicular helper cells in OSCC tumors, and the decreased plasma cells and CD8+ memory resting T cell, and increased M2 macrophage in TCGA-HNSCC tumors were also eminent. The negative correlation between SLC1A5 expression and the scores of CD8^+^ T cells, monocytes, and dendritic cells in TCGA-HNSCC tumors was confirmed by analysis using XCELL algorithm (Table S3). This algorithm also highlighted the negative correlation between SLC1A5 expression and immune score, and additional immune cell population. In GSEA analysis, the OSCC subset highly expressing SLC1A5 was positively correlated with immune regulation-associated gene sets, cell survival gene sets, and the ROS metabolic pathway. As for immune regulation, the most eminent disrupted pathways were related to the interferon response, inflammatory response, and the enrichment of immune suppression signals, particularly the IL6-Jak-Stat3 axis and IL2-Stat5 axes (Fig. 8B). We compared the gene expression of FaDu-sh-SLC1A5 with RNA sequencing data of our OSCC cohort. In our cohort data, the heatmap illustrated high expression of SLC1A5, together with increased expression of multiple genes such as SLC7A5, laminin subunit gamma 2 (LAMC2), laminin subunit beta 3 (LAMB3), metalloproteinase 9 (MMP9), and others. In concert with this finding, the sequencing of FaDu cell subclones revealed a synchronous decrease in the expression of these genes following the knockdown of SLC1A5 (Fig. 8C). The volcano plot demonstrated the significant up- or downregulated genes in the SLC1A5-high group, and SLC1A5 was the most significantly upregulated gene in both RNA sequencing data (Fig. 8D). Among these genes, the expression of LAMC2, TNF receptor superfamily member 6B (TNRSF6B; also named Decoy receptor 3, Dcr3), LAMB3, MMP9, and SLC7A5 had a positive correlation with SLC1A5 in OSCC (Fig. 8E). Except for TNFRSF6B, whose TPM data were not available in TCGA-HNSCC dataset, the expression of LAMC2, LAMB3, MMP9 and SLC7A5 was upregulated in TCGA-HNSCC tumors comparing to normal. In addition, the expression of LAMB3 was positively correlated with SLC1A5 expression in TCGA-HNSCC (not shown).

## Discussion

We identified 8 out of 11 potential glutamine transporters upregulated in OSCC or HNSCC. However, the knockdown of SLC1A5 would result in glutamine deficiency and oncogenic attenuation, highlighting the pivotal roles of SLC1A5 in glutamine uptake. Although SLC38A1 or SLC38A2 glutamine transporters are also upregulated in tumors, which may functionally compensate the SLC1A5 deficiency, the solitary knockdown of SLC1A5 resulted in cell cycle arrest in the G0/G1 phase and the blockage of S phase entry, which is consistent with previous findings in esophageal cancer, gastric cancer, and ovarian cancer [3, 22, 23].

In addition to cell cycle retardation, the present study also identifies a decrease in cell viability following the knockdown of SLC1A5. Ferroptosis results from iron-dependent lipid peroxidation, and glutamine is one of the three amino acids that make up glutathione, which functions to reduce oxidative stress [24]. Metabolic reprogramming can promote tumor chemo or radiation resistance by increasing antioxidation [25, 26]. We notify that knockdown SLC1A5 in HNSCC cells increases cleaved caspase 3 and LC3B protein expression, apoptosis and autophagy. Treatments with inhibitors a and the silencing of ATG-5 or p62 expression eliminate cell death following the knockdown of SLC1A5. Since complicated cross-talk of death programs exist in cells [27, 28], this study also identifies the concordant appearance of apoptosis, autophagy and ferroptosis, which may underlie the decreased viability in tumor cells following the SLC1A5 knockdown or cisplatin treatment. As NAC rescues program cell death associated with SLC1A5 silencing in OECM1 cells, the triggering of ROS is considered a key causative factor for cell death. Since 3-MA diminishes the apoptosis and ferroptosis associated with SLC1A5 silencing in OECM1 cells, the autophagic induction by ROS may predispose the induction of other types of programmed death. The SLC1A5 silencing renders conspicuous growth inhibition in FaDu both in vitro and in vivo, its effects on ferroptosis induction is less eminent than apoptosis or autophagy. As inhibiting SLC1A5 expression would reduce the survival and sensitize HNSCC cells to cisplatin treatment, SLC1A5 gene-silencing strategy or a chemical inhibitor may facilitate HNSCC/OSCC therapy [22].

It is the first time that SLC1A5 has been identified as the target gene of miR-125b-5p. This study discovered increased NEAT1 levels in TCGA-HNSCC tumors, with NEAT1 knockdown leading to a reversal of miR-125b-5p expression. The upregulation of SLC1A5 by promoter activation has been reported in a previous study [29]. The dCas9-SAM experiments in this study confirm the promoter activation in driving SLC1A5 upregulation. Besides, the sponging of miR-125b-5p by NEAT1 transcript may result in the epigenetic upregulation of the SLC1A5 protein in HNSCC/OSCC. The findings suggest that SLC1A5 blockage via its upstream regulators could also be a crucial strategy for HNSCC/OSCC interception [30].

The expression of SLC1A5 confers advantages in glutamine uptake in triple-negative breast cancer cells, accompanied by the attenuation of CD8^+^ and CD4^+^ T cell function [31]. This study identified decreased CD8^+^ T cells and follicular helper T cells, likely due to the lack of monocytes in presenting tumoral antigens in HNSCC tumors with high SLC1A5 expression. In the hallmark GSEA analysis, we also found that E2F targets, MYC target V1, MYC target V2, G2M checkpoint, and MTORC1 signaling pathway were significantly upregulated, with a normalized enrichment score of over 1.5 in the highly expressed SLC1A5 group. To determine which genes are more important in the SLC1A5-high group, we further analyzed the gene expression changes in these hallmark gene sets in the FaDu-sh-SLC1A5 group and our cohort. In the cohort group, ECM-related genes such as LAMC2, LAMB3, MMP9, and others were upregulated, along with increased expression of SLC1A5 and SLC7A5. Previous studies have indicated that LAMC2 regulates the TGF-β signaling pathway and the integrin β1- and ZEB1-dependent pathways to promote cancer progression [32, 33]. However, LAMB3 activates the PI3K/Akt signaling pathway to support cancer cell survival, invasion, and metastasis [34]. In the downregulated gene part, immune-regulated genes such as GZMB, DIO2, and CD8A were decreased, which is consistent with our algorithmic analysis. As a large different immune cell population seems to decrease in tumors exhibiting high SLC1A5 expression, the roles of SLC1A5 in modulating microenvironmental or immune profiles deserve comprehensive investigation [4]. TNFRSF6B is involved in the apoptosis pathway, but it can activate NF-κB to support tumor cell survival [35]. Conversely, the expression levels of these genes were completely reversed in the FaDu-sh-SLC1A5 group. It is worth noting that the CDKN1A gene, which encodes the protein p21 and plays critical roles in DNA damage repair, cell cycle inhibition, and tumor suppression, was significantly upregulated in the FaDu-sh-SLC1A5 group. CDKN1A-high patients usually have a better overall survival rate. In our cohort, the volcano plot results depict a similar pattern, with increased expression of LAMC2, LAMB3, TNFRSF6B, and MMP9, which positively correlated with SLC1A5 expression. Therefore, SLC1A5-high tumor cells undergo substantial changes in cell proliferation and cell cycle-associated genes to promote HNSCC progression.

Previous studies specified the modulation of SLC1A5 on the oncogenesis, glutamine metabolism, ROS induction, apoptosis, autophagy, and CD8 + T infiltration in HNSCC/OSCC cells or tumors [7–9, 12]. This study elaborates NEAT1/miR-125b-5p as SLC1A5 upstream regulators and identifies potential downstream effectors of SLC1A5 for the neoplastic or immune modulation in HNSCC/OSCC. This study also specifies the association between SLC1A5 downregulation and the induction of oxidative stress and ferroptosis in HNSCC/OSCC, which may infer therapeutic implications. Although the analysis of transcriptional states of SLC1A5 does not yield prognostic implications, the unequivocal discoveries on the influences of SLC1A5 on the infiltration of multiple immune cell components in the TME of HNSCC/OSCC may substantiate the potential indication of immune therapy.

## Materials and methods

### Samples

A total of 55 OSCC patients’ tumor tissue and 30 paired adjacent normal tissue samples were analyzed in this study (Table S4). The sampling of patients and data collection was approved by the institutional review board in Taipei Mackay Hospital (IRB approval no.: 18MMHIS187e and 20MMHIS441e). Informed consent was obtained from patients before sampling.

### Cell culture

Human head and neck squamous cell carcinoma (HNSCC) cell lines FaDu (HTB-43^TM^, ATCC, Manassas, VA, USA) and OECM1 [36], Lenti-X™ 293 T Cell Line (293 T, 632180, Clonetech, Mountain View, CA, USA), and RetroPack^TM^ PT67 cell (PT67, 631510, Clonetech) were used. The FaDu cells were maintained in DMEM/F-12 (12500-062, Gibco, Carlsbad, CA, USA); OECM1 cells in RPMI 1640 (31800-022, Gibco), and 293 T as well as PT67 cells in DMEM (12100-046, Gibco). All media contained a supplement of 10% fetal bovine serum (FBS, 10437-028, Gibco), 2 mM glutamine (Gibco) and antibiotics (BII03-033-1B, Sartorius, Göttingen, Germany).

### Generation of constructs

For the reporter assay, the SLC1A5 3’ untranslated region (UTR) was obtained by amplification of sequences using primers listed in Table S5. Then, the mutated sequence was acquired using a site-directed mutagenesis strategy [37]. The wild-type and mutated SLC1A5 3’ UTR cDNA were cloned into pMIR-REPORT™ miRNA Expression Reporter Vector (AM5795, Invitrogen, Waltham, MA, USA). For endogenous SLC1A5 overexpression, the SLC1A5 activated sequence “SLC1A5-dCas9-SAM-S and SLC1A5-dCas9-SAM-AS” and the scramble sequence “NC-dCas9-SAM-S and NC-dCas9-SAM-AS” were mixed and annealed, then cloned into the sgRNA (MS2) vector (# 61424, Addgene, Watertown, MA, USA) to generate SLC1A5 promoter target gRNA (SLC1A5-gRNA) and scramble gRNA (scr-gRNA) expression vector [38]. The guide RNA sequences are listed in Table S6.

### Establishment of HNSCC cell subclones

The pCMVΔR8.91, pMD.G., sh-SLC1A5, and sh-TCR1 vectors were obtained from the National Core Facility for Biopharmaceuticals for lentivirus packing. The sh-SLC1A5 or sh-TCR1 expressed lentivirus was used to infect HNSCC to establish stable clones, respectively [39]. The HNSCC subclones were used within 20 passages. The lentiSAM v2 (# 92062, Addgene) and lentiMPH v2 (# 89308, Addgene) were separately transfected into PT67 cells to pack retrovirus. OECM1 cells were first infected with lentiSAMv2 virus to express dCas9-VP64 fusion protein, followed by selection with 8 μL/mL blasticidin (15205, Sigma-Aldrich, St. Louis, MO, USA) for one week. The selected cells were re-infected with lentiMPH v2 virus and selected with 100 µg/mL hygromycin b (10843555001, Roche, Basel) for a week to establish OECM1-dCas9-SAM cells. All transfection processes followed the manual of Lipofectamine™ 3000 Transfection Reagent.

### The mimic, inhibitor, si-RNA, and their scramble treatment

The doses of miR-125b-5p mimic and inhibitor were validated in our previous study [39]. For the miR-125b-5p/SLC1A5 axis verification, cells were treated with mirVana™ miRNA Mimic Negative Control #1 (Scr mimic, 4464059, Invitrogen), miR-125b-5p mimic (MC10148, Invitrogen), and SLC1A5 wild-type 3’UTR reporter vector, SLC1A5 mutant 3’UTR reporter vector, or reporter vector alone to perform reporter assay. For the verification of the relevance between miR-125b-5p and SLC1A5, mirVana™ miRNA Inhibitor Negative Control #1 (4464076, Invitrogen) or hsa-miR-125b-5p inhibitor (MH10148, Invitrogen) was used for treatment for 24 hours. In rescue experiments, cells were treated with 60 nM si-p62 (si-sequestosome 1, sc-29679, Santa Cruz Biotech, Santa Cruz, CA, USA), si-ATG5 (sc-29679, Santa Cruz Biotech), and Control siRNA-A (si-scramble, sc-37007, Santa Cruz Biotech). To verify the correlation between NEAT1 and miR-125b-5p, cells were treated with 100 nM si-NEAT1 (n272461, Invitrogen), si-SLC1A5 (s12916, Invitrogen), or Silencer™ Select Negative Control No. 1 siRNA (4390843, Invitrogen). To rescue the SLC1A5 expression being affected by NEAT1-miR-125b-5p, cells were treated with 100 nM si-NEAT1 with or without 100 nM mirVana™ miRNA Inhibitor Negative Control #1 or hsa-miR-125b-5p inhibitor. Transfection was used for OECM1 cells, while FaDu cells underwent electroporation according to the protocol outlined in the Cell Line Nucleofector™ Kit V (VCA-1003, Lonza, Basel, Switzerland) and utilizing the Nucleofector^TM^ 2b Device (AAB-1001, Lonza).

### Quantitative real-time polymerase chain reaction (qPCR)

Total RNA was extracted using TriPure™ Isolation Reagent (11667165001, Roche) and reverse transcribed using MMLV High-Performance Reverse Transcriptase (RT80125K, LGC Biosearch Tech, London, UK) or MicroRNA Reverse Transcription Kit (4366596, Applied Biosystems, Waltham, MA, USA) to yield cDNA. qPCR reactions were performed in duplicate. RNU6B and GAPDH served as the internal controls [36].

### Western blot

An amount of 30 µg protein was used for western blot analysis. The primary antibodies and secondary antibodies are listed in Table S7. The signals of tested proteins were normalized to GAPDH to yield the expression values. The detailed procedures followed our previous protocol [39].

### Phenotypic and biochemical assays

The trypan blue exclusion assay was used to measure cell viability every 24 hours to generate the growth curve of cells. The cell viability of cells following treatment with cisplatin (232120, Sigma-Aldrich) was analyzed by MTT assay to acquire a drug resistance index [40]. Cell cycle analysis was performed on HNSCC subclones with SLC1A5 knockdown [40]. Cell migration and invasion assays followed the previous protocol [36]. The glutamine or GSH/GSSG ratio measurement followed the Glutamine-Glo kit (J7021, Promega, Madison, WI, USA) and GSH/GSSG-Glo kit (V6611, Promega) protocols provided by the supplier. For the tumorigenesis assay, 10^6^ FaDu-sh-TCR1 or FaDu-sh-SLC1A5 cells were injected subcutaneously into the flanks of randomly grouped 6–8 weeks nude mice (National Laboratory Animal Center, Taipei, Taiwan). Three nude mice were used in each group. Tumor volume calculation uses the formula 1/2 × longest diameter × (shortest diameter)^2^. The animal study was approved by the Institutional Animal Care and Use Committee (IACUC) of National Yang Ming Chiao Tung University (approval no. 1100422). In rescue assay, the knockdown SLC1A5 HNSCC subclones were treated with 5 µM Z-VAD-FMK (V116, Sigma-Aldrich), 5 µM 3-Methyladenine (3-MA, 189490, Sigma-Aldrich), 5 µM Ferrostatin-1 (SML0583, Sigma-Aldrich), or Dimethyl sulfoxide (DMSO, C6164, Merck, Darmstadt, Germany) for 48 hours, and cell numbers were then counted by trypan blue exclusion assay. To detect reactive oxidative species (ROS), cells were stained with 5 µM CellROX Deep Red Reagent (C10422, Invitrogen) and analyzed with flow cytometry. To detect lipid peroxidation, cells were seeded onto an x-well Lumox slide (94.6150.401, SARSTEDT, Nümbrecht, Germany). The next day, they were treated with 100 nM si-SLC1A5 for 24 hours. The cisplatin treatment was administered at the third day for another 24 hours. Before detection, the culture medium was replaced with 1 µM BODIPY 581/591 C11 (D3861, Invitrogen), and the cells were incubated for 1 hour. Fluorescent images were captured using a confocal microscope (LSM880, Zeiss, Oberkochen, Germany). The OECM1-sh-TCR1 or FaDu-sh-TCR1 cells treated with DMSO were used as a reference to calculate cell viability as a percentage.

### Reporter assay

The reporter assay was performed using the Dual-Luciferase^®^ Reporter Assay System (E1910, Promega) according to the protocol. Renilla luciferase activity was used as the transfection efficiency control normalized with firefly luciferase activity [36].

### Apoptosis, autophagy, and ferroptosis detection by flow cytometry

OECM1 subclones were treated with cisplatin for 24 hours, while FaDu subclones were treated for 48 hours at their respective IC_50_ dosages. Subsequently, cells were harvested along with the supernatant. Propidium iodide solution (P4864, Sigma Aldrich) was used to stain the cells to assess apoptosis. The cell-permeable fluorescent molecule “DAPGreen” (D676, DOJINDO Lab., Kumamoto) has a high affinity with LC3B and could be incorporated into autophagosomes. The fluorescent probe FerroOrange (F374, DOJINDO Lab.) indicates ferroptosis and interacts with intracellular Fe^2+^. All of the fluorescent molecule staining processes were followed according to the manual. To inhibit ROS, cells were treated with 1 mM or 20 mM N-Acetyl-L-cysteine (NAC, A7250, Sigma-Aldrich) dissolved in ddH_2_O for 1 hour prior to experiments [41].

### RNA sequencing and bioinformatic processing

Qualified RNA-Seq libraries of tissues and cells being constructed were subjected to RNA sequencing using the NovaSeq 6000 system (Illumina, San Diego, CA, USA) or the NextSeq 550 system (SY-415-1002, Illumina). The clean reads of transcripts were transformed to transcripts per million (TPM). The TPM values of the HNSCC dataset were downloaded from TCGA (http://cancergenome.nih.gov/, project ID - TCGA-HNSC). The medium TPM value of SLC1A5 in OSCC tumors, and the medium or mean TPM values of SLC1A5 in TCGA-HNSCC are used as cutoffs to designate high (H) or low (L) expression in SLC1A5. Gene Set Enrichment Analysis (GSEA) was conducted using GSEA software (version 4.2.3) and ggplot2 (version 3.4.0) for plotting. ImmuneScore was determined by accessing the Cibersortx portal (https://cibersortx.stanford.edu) and XCELL portal (https://xcell.ucsf.edu). Potential miRNA-SLC1A5 targeting was predicted by accessing database portals, including TargetScan, TarBase, miRGator, miRDB, miRanda, and mirDIP.

### Statistical analysis

All the data were shown in mean ± standard error (SE). Mann-Whitney test, *t*-test, ANOVA test, and correlation analysis were used to analyze correlations across variants. The analyses were performed using GraphPad Prism v7.0 software (GraphPad) and CytExpert Software (version 2.4). *P* < 0.05 indicates a statistically significant difference.

### Supplementary information

Supplementary

## Data Availability

All the data can be found either in the main text or the supplementary materials.
