# Supplementary material for: The disruption of NEAT1-miR-125b-5p-SLC1A5 cascade defines the oncogenicity and differential immune profile in head and neck squamous cell carcinoma
Source: Cell Death Discov. 2024 Sep 3;10:392. doi: 10.1038/s41420-024-02158-1 (PMC11369192; doi:10.1038/s41420-024-02158-1)
Supplement: Supplementary file 1 — Supplementary [file 41420_2024_2158_MOESM1_ESM.docx]

**Supplementary Tables**

Table S1. The expression of SLC1A family members, SLC38A1 and SLC38A2 in TCGA-HNSCC tumors

| **SLC** | **Folds (HNSCC/normal)** | ***P*** | **Correlation with SLC1A5** |
| --- | --- | --- | --- |
| **1A5** | 1.83 | 4.60E-17 | reference |
| **1A1** | 0.39 | 4.90E-12 | ***, positive |
| **1A2** | 0.84 | 0.0093 | no |
| **1A3** | 2.32 | 0.034 | no |
| **1A4** | 2.34 | 1.60E-13 | no |
| **1A6** | 1.35 | 0.028 | no |
| **1A7** | 0.37 | 3.70E-07 | no |
| **38A1** | 1.48 | 8.80E-06 | ****, positive |
| **38A2** | 1.62 | 1.00E-07 | no |

***, *P* <0.001, ****, *P* <0.0001

Table S2. The expression of other potential glutamine transporters and their correlation with SLC1A5 expression in TCGA-HNSCC tumors

| **SLC** | **Folds (HNSCC/normal)** | ***P*** | **Correlation with SLC1A5** |
| --- | --- | --- | --- |
| 1A5 | 1.83 | 4.60E-17 | reference |
| 6A14 | 0.06 | 0.0031 | ****, negative |
| 6A19 | not available |  |  |
| 7A5 | 2.56 | 3.00E-14 | no |
| 7A8 | 3.91 | 4.80E-11 | **, negative |
| 38A3 | 0.11 | 7.60E-30 | ****, negative |
| 38A5 | 3.53 | 8.60E-06 | ***, negative |
| 38A7 | 2.26 | 3.40E-36 | no |
| 38A9 | 1.43 | 6.20E-07 | no |

**, *P* <0.01, ***, *P* <0.001, ****, *P* <0.0001

Table S3. The correlation between immune cell score and SLC1A5 expression analyzed with XCELL algorithm in TCGA-HNSCC tumors

| **Cell population** | ***r*** | ***P*** |
| --- | --- | --- |
| T cell CD8+ naïve | -0.113 | 0.0101 |
| T cell CD8+ | -0.102 | 0.0199 |
| Monocyte | -0.304 | <0.0001 |
| Myeloid dendritic cell | -0.158 | 0.0003 |
| Myeloid dendritic cell activated | -0.144 | 0.001 |
| T cell CD4+ central memory | -0.384 | <0.0001 |
| T cell CD4+ effector memory | -0.201 | <0.0001 |
| Endothelial cell | -0.170 | 0.0001 |
| Macrophage M2 | -0.094 | 0.0321 |
| Mast cell | -0.177 | <0.0001 |
| Neutrophil | -0.228 | <0.0001 |
| Plasmacytoid dendritic cell | -0.151 | 0.0005 |
| B cell plasma | -0.111 | 0.0113 |
| T cell regulatory | -0.116 | 0.0083 |
| Common lymphoid progenitor | 0.107 | 0.0145 |
| T cell CD4+ Th1 | 0.104 | 0.0177 |
| T cell CD4+ Th2 | 0.228 | <0.0001 |
| Immune score | -0.144 | 0.001 |
| Microenvironment score | -0.144 | 0.001 |

Yellow box, the immune cell population identified in Fig. 8A; red box, positively correlated; blue box, negatively correlated

Table S4. Clinicopathological parameters of OSCC samples

| Gender |  |  |
| --- | --- | --- |
|  | Male | 53 |
|  | Female | 2 |
| Age |  |  |
|  | ≧ 60 | 30 |
|  | < 60 | 25 |
| T |  |  |
|  | 1-3 | 15 |
|  | 4 | 40 |
| N |  |  |
|  | 0 | 31 |
|  | 1-3 | 24 |
| Stage |  |  |
|  | I-III | 13 |
|  | IV | 42 |
| Grade |  |  |
|  | 1 | 40 |
|  | 2, 3 | 15 |

Table S5. PCR primer used to amplify 3'UTR sequence in SLC1A5

| Primer | Sequence |
| --- | --- |
| SLC1A5 3’UTR wild-type forward | 5’-TATAGAGCTCACCCCGGGAGGGACCTTC-3’ |
| SLC1A5 3’UTR wild-type reverse | 3’-TTAGCAATAAAATTGAGTGTCAACTATTTAAGCTTGCG-5’ |
| SLC1A5 3’UTR mutant forward | 5’-CTGCCTGGCCTCCCCTTAGATCTAACGCAGGTCACAG-3’ |
| SLC1A5 3’UTR mutant reverse | 5’-GTGACCTGTGACCTGCGTTAGATCTAAGGGGAGGCCA-3’ |

Table S6. guide RNA sequence

| gRNA name | sequence |
| --- | --- |
| SLC1A5-dCas-SAM-S | 5’-CACCGTAATACCCATCTCCAGGAGC-3’ |
| SLC1A5-dCas-SAM-AS | 5’-AAACGCTCCTGGAGATGGGTATTAC-3’ |
| NC-dCas-SAM-S | 5’-CACCGTATTACTGATATTGGTGGG-3’ |
| NC-dCas-SAM-AS | 5’-AAACCCCACCAATATCAGTAATAC-3’ |

Table S7. Antibodies used in this study

| Antibody | Host | Cat. No | Supplier |
| --- | --- | --- | --- |
| Cleaved Caspase 3 | Rabbit | #9661S | Cell Signaling |
| GAPDH | Mouse | Sc-32233 | Santa Cruz Biotech |
| LC3B | Rabbit | #2775S | Cell Signaling |
| SLC1A5 | Rabbit | #5206S | Cell Signaling |
| Goat Anti-Mouse |  | AP124P | Merck Millipore |
| Goat Anti-Rabbit |  | AP132P | Merck Millipore |

**Supplementary figures**

**
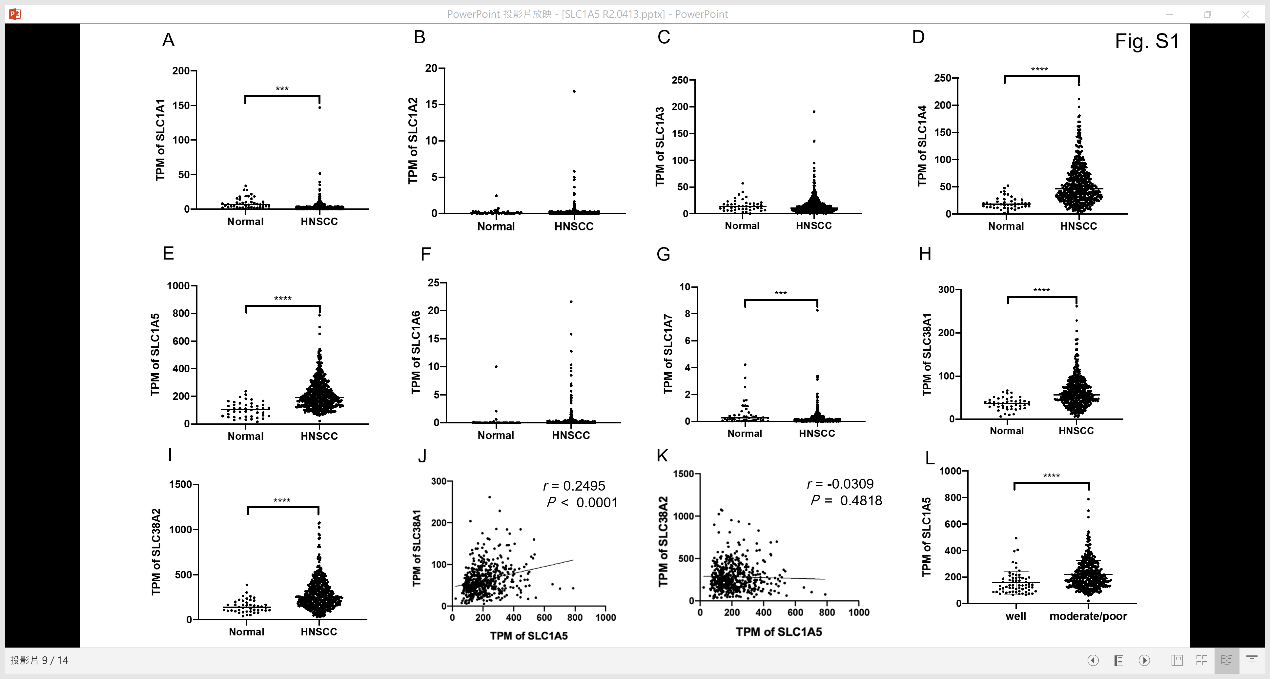
**

Figure S1.

Analysis of TCGA-HNSCC RNA sequencing data. **A – I.** The TPM of the SLC1A family, SLC38A1, and SLC38A2 in normal tissues (*n* = 44) and HNSCC tumor tissue (*n* = 522). **J, K.** The correlation between SLC1A5 and SLC38A1, or SLC38A2. **L.** The **c**orrelation between SLC1A5 and tumor differentiation (well-differentiated, *n* = 62, moderately/poorly-differentiated, *n* = 436). Unpaired *t*-test or correlation analysis. *** and **** represent *P* < 0.001 and *P* < 0.0001, respectively.


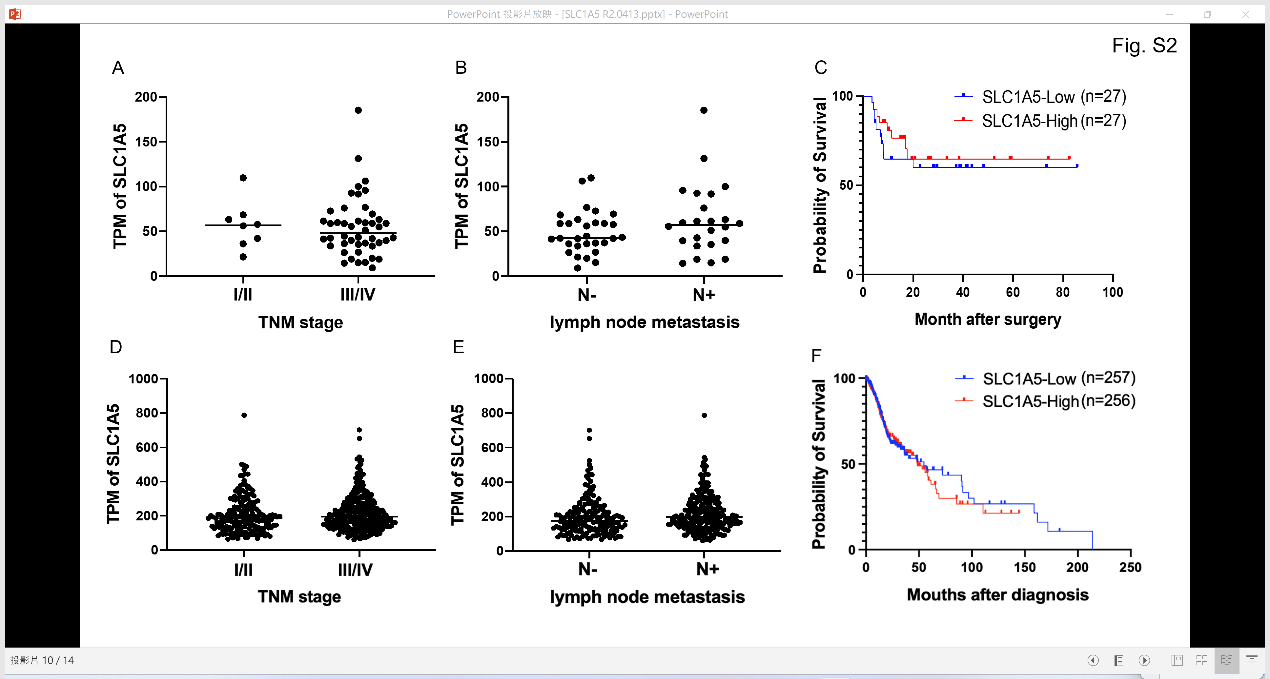


Figure S2.

The association between clinical parameters and SLC1A5 TPM. **A – C,** OSCC tumors. **D – F,** TCGA-HNSCC tumors. **A, D,** clinical stage. **B, E,** lymph node metastasis. **C, F,** Kaplan-Meier survival curve. **A.** I/II, *n* = 8; III/IV, *n* = 47. **B,** N-, *n* = 32; N+, *n* = 23. **C,** SLC1A5-Low, *n* = 27; SLC1A5-High, *n* = 27. One sample having questionable follow-up data is eliminated for survival analysis. **D**, I/II*, n* = 185; III/IV, *n* = 312. **E**, N-, *n* = 176; N+, *n* = 244. **F,** SLC1A5-Low, *n* = 257; SLC1A5-High, *n* = 256. The medium TPM value of SLC1A5 in tumors separates SLC1A5-Low and SLC1A5-high subsets. Mann-Whitney test or Kaplan-Meier analysis. No statistically significant difference is found across the comparison of groups.


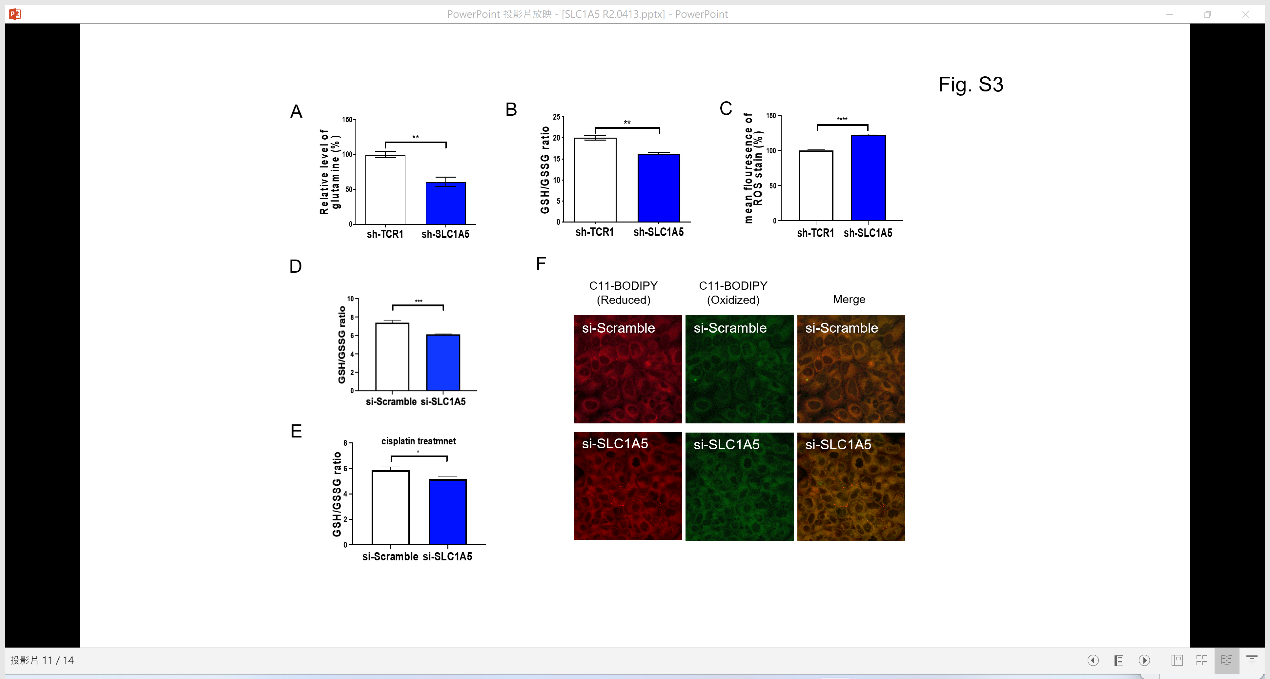


Figure S3.

The ROS-associated states in FaDu cells following the knockdown of SLC1A5. **A - C.** The glutamine, GSH/GSSG ratio, and ROS detected by CellROX Deep Red Reagent staining in cell subclones, respectively. **D - F.** Cells transfected with si-Scramble or si-SLC1A5. **D, E.** Cells treated with 15.7 µM cisplatin for 48 hours. **D, E.** GSH/GSSG ratio. **F.** Confocal microscopy to illustrate the C11-BODIPY-labelled polyunsaturated fatty acids in cells. Left panels, reduced form (red); Middle panels, oxidized form (green); Right panels, merged images. Mann-Whitney test. *, ** and ***, *P* < 0.05, *P* < 0.01 and *P* < 0.001, respectively.


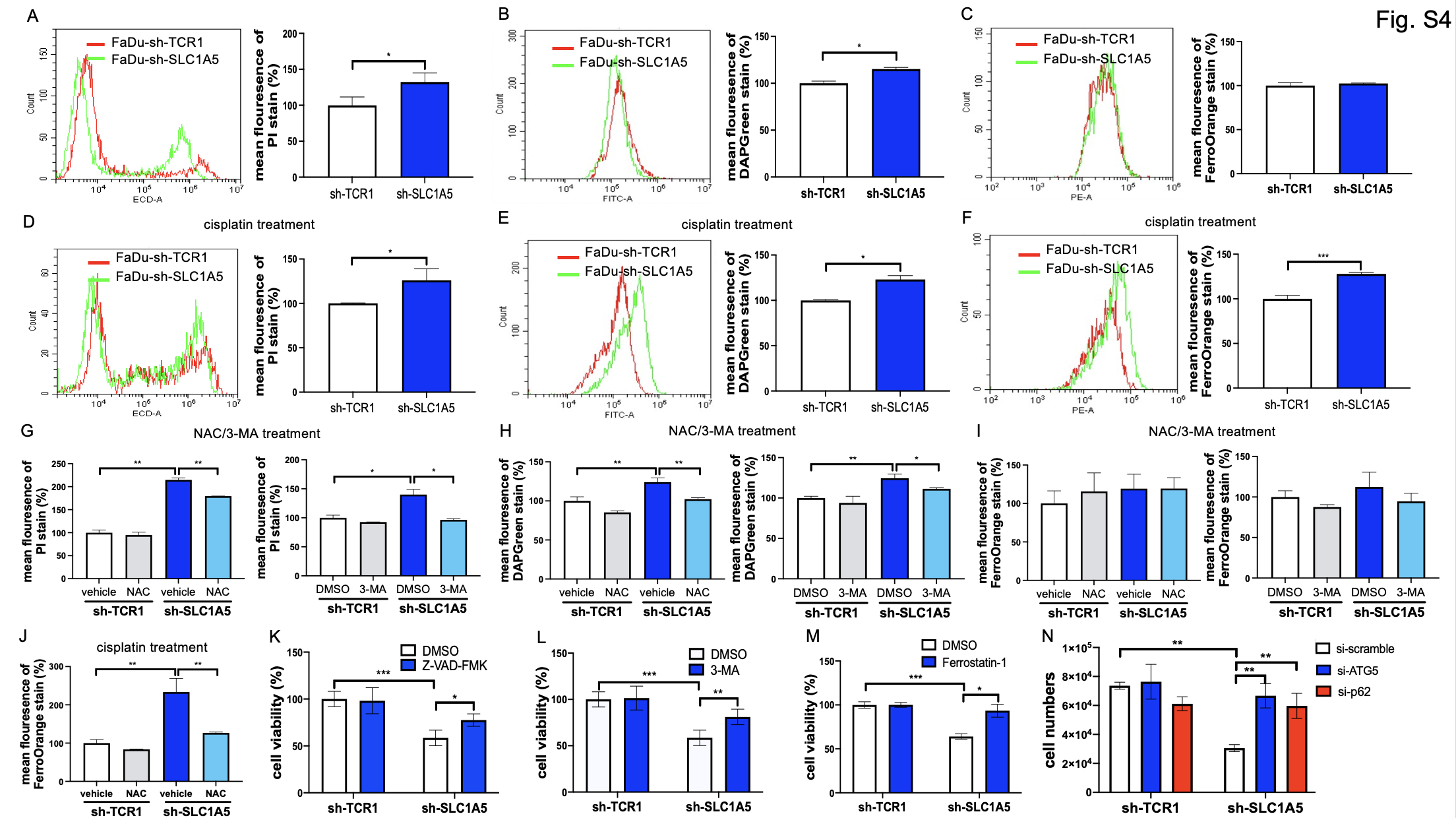


Figure S4.

Knockdown of SLC1A5 and cell death in FaDu cell subclones. **A - C** and **D - F.** Flow cytometry analysis of cell subclones without (**A - C)** or with the treatment of 15.7 µM cisplatin for 48 hours **(D - F)**, respectively. **A, D.** PI stain as an indicator of apoptosis. **B, E.** DAPGreen is the dye of autophagosome (LC3B). **C,** **F.** FerroOrange is a fluorescent probe for ferroptosis (intracellular Fe^2+^). Lt, histograms, Rt, quantitation of the positive cells. **G - I**. The quantitation of PI staining, DAPGreen staining, and FerroOrange staining in cell subclones following NAC or 3-MA treatment, respectively. Lt, 1 mM NAC treatment, Rt, 5 µM 3-MA treatment. **J.** The quantitation of FerroOrange staining in cell subclones following 15.7 µM cisplatin treatment for 24 hours without or with 20 mM NAC pretreatment 1 hour. **K-N.** Rescue assay of cell viability. Treatment with 5 µM Z-VAD-FMK (**K**), 3-MA (**L**), Ferrostatin-1 (**M**), or 60 nM si-Scramble, si-ATG5, and si-p62 (**N**) is performed in cell subclones for 48 hours, respectively. These inhibitors or siRNAs reverse the decreased cell viability associated with the knockdown of SLC1A5. Mann-Whitney test. *, ** and ***, *P* < 0.05, *P* < 0.01 and *P* < 0.001, respectively.


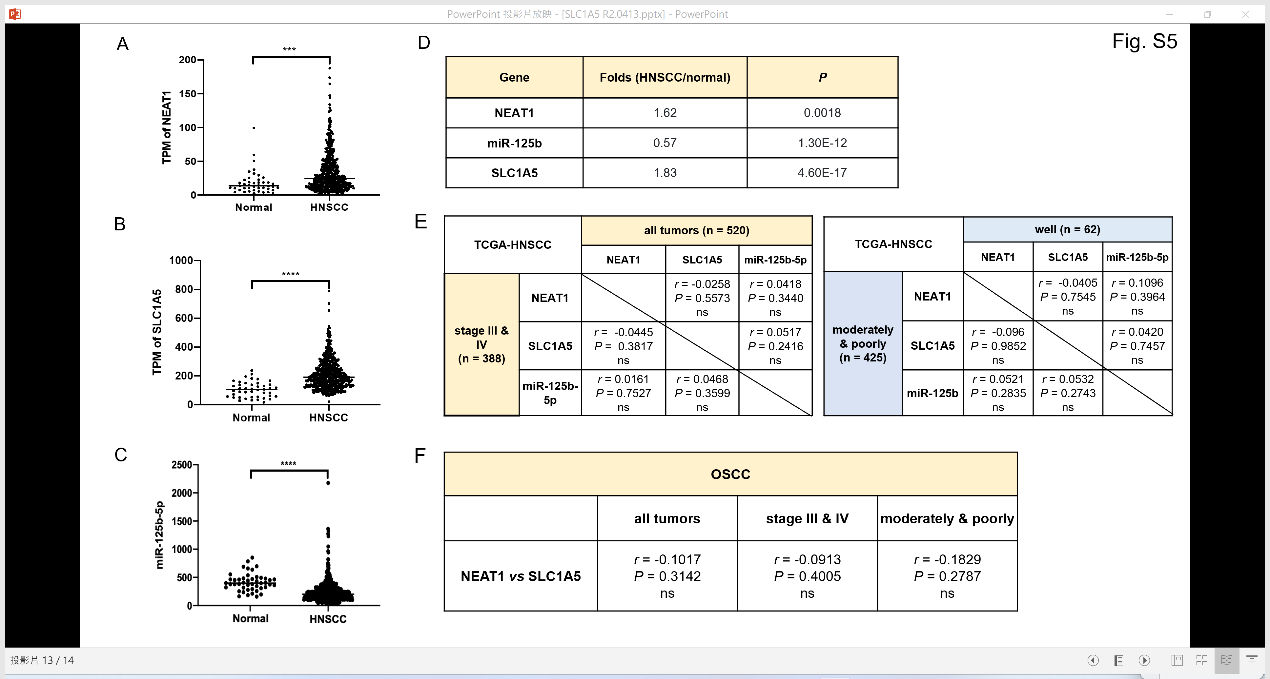


Figure S5.

The expression of NEAT1, miR-125b-5p, and SLC1A5 in TCGA-HNSCC tumor datasets or our OSCC cohort. **A - E.** TCGA-HNSCC tumors. **F.** our OSCC tumors. **A.** NEAT1, **B.** miR-125b-5p, **C.** SLC1A5. Unpaired *t*-test. *** and **** represent *P* < 0.001 and *P* < 0.0001, respectively. **D.** Fold changes and *P* values of data in (**A - C**). **E.** Analysis of correlation of NEAT1, SLC1A5, and miR-125b-5p in TCGA-HNSCC tumors according to all tumors or late-stage tumors (Lt) and tumor grades (Rt). **F.** Analysis of the correlation between NEAT1 and SLC1A5 in OSCC tumors according to all late-stage tumors or tumor grades. *r*, correlation coefficient.


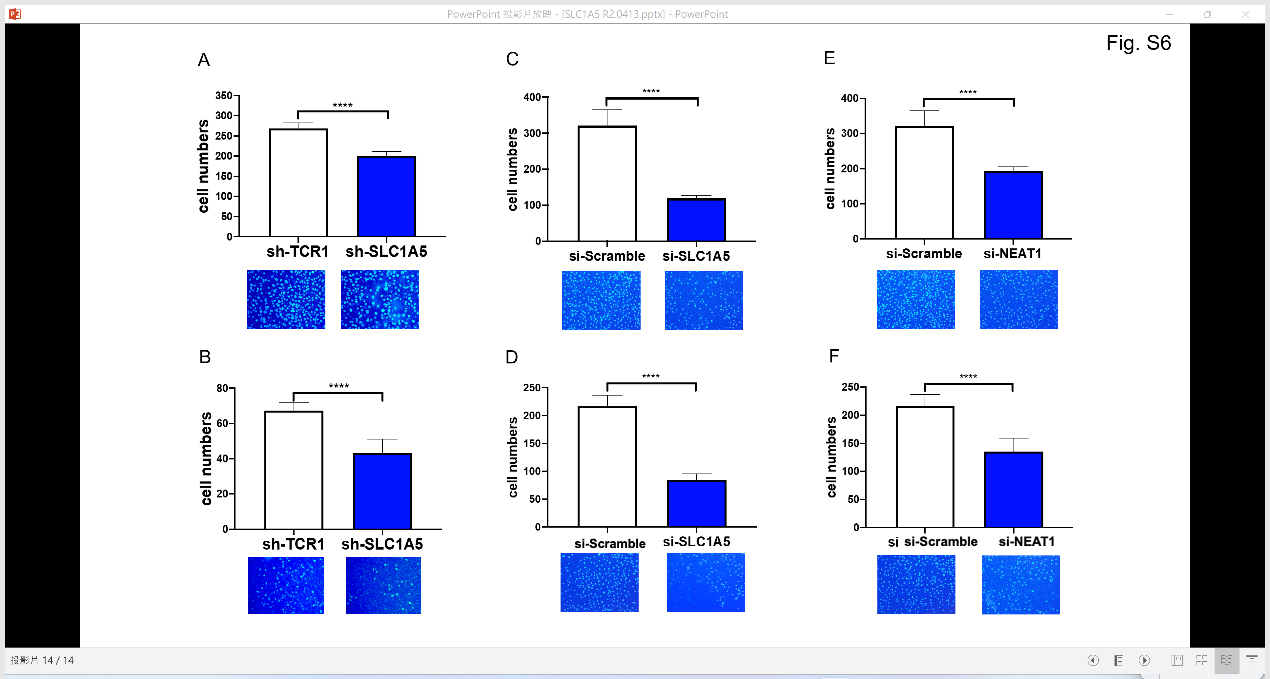


Figure S6.

The migration and invasion assay of FaDu cells following the knockdown of SLC1A5 or NEAT1. **A, B.** cell subclones. **C, D,** and **E, F.** knockdown of SLC1A5 and NEAT1 using siRNA, respectively. The dose of si-Scramble, si-SLC1A5, and si-NEAT treatment is 60 or 100 nM. **A, C, E.** migration assay. **B, D, F.** invasion assay. Mann-Whitney test. ****, *P* < 0.001.
